# Attending a Biopsychosocially Focused Buprenorphine Training Improves Clinician Attitudes

**DOI:** 10.3389/fpsyt.2021.639826

**Published:** 2021-08-02

**Authors:** Holly Ann Russell, Brian Smith, Mechelle Sanders, Elizabeth Loomis

**Affiliations:** ^1^Department of Family Medicine, University of Rochester Medical Center, Rochester, NY, United States; ^2^University of Rochester School of Nursing, Rochester, NY, United States; ^3^United Memorial Medical Center, Batavia, NY, United States

**Keywords:** substance use disorder, opioid use disorder, stigma, biopsychosocial, medications for opioid use disorder

## Abstract

**Objective:** Substance use disorders remain highly stigmatized. Access to medications for opioid use disorder is poor. There are many barriers to expanding access including stigma and lack of medical education about substance use disorders. We enriched the existing, federally required, training for clinicians to prescribe buprenorphine with a biopsychosocial focus in order to decrease stigma and expand access to medications for opioid use disorder.

**Methods:** We trained a family medicine team to deliver an enriched version of the existing buprenorphine waiver curriculum. The waiver training was integrated into the curriculum for all University of Rochester physician and nurse practitioner family medicine residents and also offered to University of Rochester residents and faculty in other disciplines and regionally. We used the Brief Substance Abuse Attitudes Survey to collect baseline and post-training data.

**Outcomes:** 140 training participants completed attitude surveys. The overall attitude score increased significantly from pre to post-training. Additionally, significant changes were observed in non-moralism from pre-training (*M* = 20.07) to post-training (*M* = 20.98, *p* < 0.001); treatment optimism from pre-training (*M* = 21.56) to post-training (*M* = 22.33, *p* < 0.001); and treatment interventions from pre-training (*M* = 31.03) to post-training (*M* = 32.10, *p* < 0.001).

**Conclusion:** Increasing medical education around Opioid Use Disorder using a Family Medicine trained team with a biopsychosocial focus can improve provider attitudes around substance use disorders. Enriching training with cases may improve treatment optimism and may help overcome the documented barriers to prescribing medications for opioid use disorder and increase access for patients to lifesaving treatments.

## Introduction

Substance use disorders (SUDs) are among the most stigmatized conditions in the US and around the world (1). Furthermore, stigma against people with opioid use disorder (OUD) and other SUDs affect the frequency with which people are offered effective treatment as well as how often they are willing to engage in care (2). Clinicians explicitly acknowledge treatment pessimism and negative stereotypes about patients with OUDs as a barrier to offering care (3, 4). The recent COVID-19 pandemic has highlighted the shortfalls of the current behavioral health treatment system and the additional risks for patients with underlying mental health issues (5). Efforts to increase the understanding of substance use disorders as a chronic disease have highlighted the underlying neurobiological changes and focused on biomedical approaches to treatment. At times this approach may have furthered the stigma surrounding opioid use disorder (6). The term “Medication Assisted Therapy” or MAT is different than the terminology surrounding pharmacotherapy for any other chronic disease. For example, we would never describe the use of metformin for diabetes as medication assisted treatment or say medication assisted treatment when advocating for diuretics for blood pressure management.

Medications for opioid use disorder (MOUD), including buprenorphine, have been shown to decrease risks of overdose and increase retention in treatment (7). Unfortunately, many patients are unable to access this treatment due to lack of providers able to prescribe the medication (8).

Many clinicians do not receive robust training around SUD treatment in undergraduate and graduate medical education (9) and medical providers frequently cite concerns related to working with patients with OUD as one of the barriers to prescribing medications for OUD thus further worsening patients' access to care (10, 11). In the United States, where this study was completed, until recently clinicians had to certify 8 h of nationally approved additional training in SUD for physicians and 24 h of additional training for Advance Practice Practioners (APPs) or see patients in a specially licensed facility to be able to prescribe MOUD. There has never been a study looking at which type and how much education is critical to improve clinicians' understanding and attitudes about SUDs. Some have argued that requiring additional training and a special license increases the stigma toward treating patients with OUD and decreases access to MOUD (12). In April 2021, the national laws changed to allow clinicians with an active DEA license to submit a notice of intent (NOI) to prescribe buprenorphine with a patient limit of 30 *without* certifying to any additional training (13).

The removal of the requirement of a certified training is an important first step to increasing access. However, in order to increase the numbers of clinicians who are actively prescribing MOUD, providers will likely need some training and education to overcome the stigma attached to substance use and to understand opioid use disorder as a chronic disease with treatment that must address the biopsychosocial needs of the patients (14). In Primary Care, our fundamental goal is to support patient disease prevention and manage understanding the interplay of biologic, social and psychiatric factors (15). Evidence from multiple continents including Europe, Australia and North America indicates that expanding access to treatment of OUD in Primary Care can decrease mortality rates without increasing the burden on clinicians (16). Substance use disorder training that focuses on expanding the comfort and knowledge of primary care clinicians to manage the long-term treatment of substance use disorders may increase access to critically needed, patient centered care for these conditions.

## Materials and Methods

### Intervention

**Figure 1:**
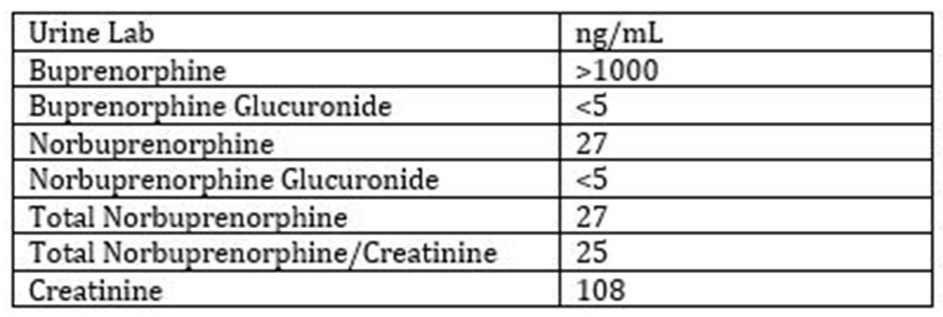
Urine drug screen (USD) results.

We received a grant to deliver nationally approved buprenorphine waiver trainings given by a primary care team at no cost across multiple settings in a 13-county region surrounding Rochester, New York. This study looked at the effect of these buprenorphine waiver trainings on clinician attitudes toward persons with SUDs. Our faculty worked with experts in the field to become certified to deliver nationally recognized trainings on all forms of OUD treatment through the American Society of Addiction Medicine (ASAM) and the American Academy of Addiction Psychiatry (AAAP). We received permission to add local data, a case that highlighted our experience from a Family Medicine perspective and anecdotes about our experience prescribing buprenorphine including how to gain acceptance from office staff and practice partners to the nationally approved waiver training slides. The biopsychosocial model acted as a framework to guide our training approach. We promoted the understanding of SUDs as a heritable chronic disease with well-documented biologic predisposition. We reviewed the frequency of mental health comorbidities in patients with SUDs and encouraged our trainees to bridge the communication gap between psychiatric care, SUD treatment and primary care. The case we added to the training highlighted the role of psychosocial stressors as potential triggers for relapse and outlined how a comprehensive biopsychosocial approach can keep patients engaged in treatment (see Table 1 for description of case and Figure 1 for the urine drug screen results). The primary training team consisted of two Family Medicine physicians and one Nurse Practioner who were trained at the University of Rochester in the Biopsychosocial model (17) and brought a combined 30 years of experience managing patients with SUDs in a primary care setting. Our focus on myth busting around concerns that treating patients with OUD would be dangerous and difficult as well as the personal and professional satisfaction that comes with adding this clinical skill helped address the biopsychosocial needs of the clinicians, and office staff.

**Table 1:** Case presentation.

**Case presentation**
• 35 year old female transferred primary care/buprenorphine management (considered stable).
• In recovery for 11 months, completed a 1 year intensive program and continuing outpatient care
• Substance Use Disorder history: Started with oral opioids after an arm fracture. Moved on to heroin. States she has used “everything.”
• At first visit she reports that she is still having cravings at 16 mg/day; requesting increase to 20 mg and states that is what she was on until she had insurance issues.
• Health history: Depression, on Citalopram
• Social History: has steady employment; recent breakup, going to family court to regain custody of kids.
• Plan: Urine Drug Screen (UDS), Labs, Contraception
Patient continues to do well and keeps follow-up appointment. Very happy to have her 2 young children home.Then a shift occurs…
• Not getting any help with kids as current partner doesn't see it as his responsibility.
• Trying to balance children/work/appointments.
• Turns out she never started her Oral Contraception prescribed at initial visit, became pregnant and then miscarried.
• UDS: See Results (Figure 1).
• Patient called by RN to come in to the office for shorter interval appointment, however she was away on a trip.
• Next visit patient adamantly denied diversion/relapse, became very angry during the visit and walked out of exam room.
For discussion: Is this patient appropriate to continue in a primary care setting? How would you approach the management of this situation?
Later in visit: Patient admitted that the inappropriate urines were not hers. She was using someone else's to hide her marijuana use.
For discussion: What are treatment options for this patient? What are risk factors for continued relapse? What would a harm reduction approach look like for this patient?

### Setting/Participants

We worked with area primary care residency programs and regional hospitals to schedule the trainings. Participants then completed the 4–4.5-h live portion of either the AAAP or ASAM mixed waiver training. Ability to participate was not restricted to specific health systems and as such participants came from 11 different health systems and both rural and urban settings.

### Instruments

We utilized the 25-item Brief Substance Abuse Attitudes Survey (BSAAS) to measure provider attitudes toward SUD (12). The survey has been previously validated among health care providers and shown good validity and reliability (12). The items are scored using a 5-point Likert Scale, with higher scores indicating a more positive attitude toward SUD. We scored the BSAAS using its published scoring metric. We calculated an overall score for BSAAS, as well as scores for the scale's five sub-scales (permissiveness, treatment intervention, non-stereotypes, treatment optimism and non-moralism). We added three questions to the BSAAS asking participant opinions regarding how effective buprenorphine treatment was for OUD, how reasonable treatment for OUD was in their practice and the degree that barriers existed in their practice to prescribe buprenorphine. We collected the survey at pre (prior to the start of a mixed, half in-person, half-online buprenorphine waiver training) and post (after the live course). We assigned each participant and a randomly assigned numerical number and retrospectively paired the surveys.

Standard demographic information was collected separately and not associated with the survey data due to concerns around protecting privacy (Table 2).

**Table 2:** Demographic and clinical characteristics.

**Characteristic**			***N***	**(%)**
Gender	(*N* = 137)	Female	62	45.3
		Male	72	52.6
		Non-binary/3rd gender	1	0.7
		Prefer not to disclose	2	1.5
Age	(*N* = 138)	25–34	74	53.6
		35–44	28	20.3
		45–54	20	14.5
		55–64	14	10.1
		65+	2	1.4
Specialty	(*N* = 138)	Addiction medicine	3	2.2
		Emergency medicine	26	18.8
		Family medicine	34	24.6
		Geriatrics	4	2.9
		Internal medicine	19	13.8
		Pediatrics	3	2.2
		Obstetrics and gynecology	2	1.4
		Other	15	10.9
		Psychiatry	26	18.8
		Palliative care	5	3.6
		Surgery	1	0.7
Years in clinical practice	(*N* = 138)	0–5 Years	61	44.2
		6–10 Years	11	8
		11–15 Years	9	6.5
		16–20 Years	9	6.5
		20–30 Years	11	8
		30+ Years	8	5.8
		I am not in clinical practice	29	21
Role	(*N* = 138)	Administrative	3	2.2
		Nurse practitioner	18	13
		Other	5	3.6
		Resident	7	5.1
		Physician	79	57.2
		Physician assistant	3	2.2
		Student	23	16.7

### Analyses

Survey completion was voluntary, with informed consent and administered across seven trainings from May 2019 to February 2020A missing value analysis was conducted to examine the amount and pattern of missing data. Descriptive statistics for the whole sample (*N* = 142) indicated that if applying listwise deletion >5% of data would be missing. We removed two cases with 50% of missing data or no post-test scores leaving an analytic sample of (*N* = 140) observations. Patterns of missingness were tested using Little's (1988) MCAR test. Data were found to be missing completely at random (MCAR) 2. Stochastic, regression-based, single imputation was used to handle all missing values for both pre- and post-test variables that comprise the BSAAS. Random error was introduced to the model to protect against narrow standard errors as a result of overfitting. Pre, post, and sub-scale scores of the BSAAS were computed and included in the model as predictors. Following imputation, subscales and total scores were recalculated.

Data management and analysis was conducted using SPSS v.26. Dependent *t*-tests were conducted on each of the five sub-scales comprising the BSAAS. Higher scores indicated more positive opinions.

### Sub-analyses

We collected demographic data separately from the attitude survey data in order to protect the privacy of our participants and to provide our funders with individual level data about who participated in the training and went on to get their “x wavier” and prescribe buprenorphine. Full demographic data was collected from 137 participants. We were not able to examine subgroup differences. The University of Rochester's Institutional Review Board (IRB) approved this study.

## Results

A total of 142 individuals completed surveys out of a potential 217 subjects. Two cases were removed due to no post-test scores, leaving an analytic sample (*N* = 140) of paired observations (65% completion rate).

### Provider Attitudes

The overall attitude score increased significantly from pre- to post-training. Additionally, significant changes were observed in non-moralism from pre-training (*M* = 20.07) to post-training (*M* = 20.98, *p* < 0.001); treatment optimism from pre-training (*M* = 21.56) to post-training (*M* = 22.33, *p* < 0.001); and treatment interventions from pre-training (*M* = 31.03) to post-training (*M* = 32.10, *p* < 0.001). No effect was observed for the scales non-stereotype or permissiveness.

In addition to the BSAAS, questions assessed participant opinions regarding how effective buprenorphine treatment was for OUD and how reasonable treatment for OUD was in their practice. Significant changes were observed for effectiveness of buprenorphine from pre-training (*M* = 4.14) to post-training (*M* = 4.5, *p* < 0.001) and for how reasonable treatment for OUD is in their practice from pre-training (*M* = 3.87) to post-training (*M* = 4.24, *p* < 0.001) (see Table 3 for full results).

**Table 3:** Summary of pre- and post-training results.

	**Pre-training**		**Post-training**				**95% CI**	
	**M**	**SD**	**M**	**SD**	***t (df)***	***P***	**Lower**	**Upper**
Non-Moralism	20.07	2.60	20.98	2.79	5.89 (139)	<0.001	0.61	1.22
Treatment optimism	21.56	2.45	22.33	2.30	5.27(139)	<0.001	0.48	1.06
Non-stereotype	12.07	1.70	12.12	1.74	0.49(139)	0.62	−0.17	0.28
Treatment interventions	31.03	3.17	32.10	3.35	4.97(139)	<0.001	0.65	1.50
Permissiveness	10.35	2.42	10.33	2.97	0.09(139)	0.93	−0.35	0.32
^*^Full BSAAS	95.07	7.47	97.87	7.83	6.42(139)	<0.001	1.94	3.67
Effectiveness of Buprenorphine	4.14	0.72	4.50	0.71	5.57(139)	<0.001	0.23	0.48
Treatment for OUD in your practice	3.87	0.90	4.24	0.70	4.75(139)	<0.001	0.22	0.52

*M, mean; SD, standard deviation; t, t-value from the dependent t-test; df = degrees of freedom; p = significance (two-tailed, p < 0.05); CI, confidence interval.*

* *BSAAS: https://medicine.yale.edu/sbirt/curriculum/modules/medicine/brief_substance_abuse_attitude_survey_100733_284_13474_v1.pdf*.

## Discussion

The results of this preliminary study show that attending a biopsychosocially focused, buprenorphine waiver training delivered by a primary care team can significantly change attitudes toward patients with substance use and improve treatment optimism. Strengths of this study include its relatively large sample size as compared to other studies looking at provider attitude changes and the fact that we were able to pair our pre-post survey results. Additionally, our study includes results from different levels of training, multiple specialties and types of clinicians, indicating that change in attitude is not limited by individual characteristics. Previous studies have shown resident attitudes decline toward patients with substance use during residency (18), but that exposure to additional training in addiction can improve attitudes toward persons with SUD (19, 20). A recent study of medical students indicated that traditional buprenorphine waiver training alone is not be enough to decrease stigma (21). A biopsychosocial focus may be the element that can help improve clinician attitudes toward this patient population.

Federal regulations previously required 8 h of training for physicians and 24 h of training for Advance Practice Practitioners in order to prescribe buprenorphine for OUD. The authors feel that the value of attending the training is in understanding more about the nature and course of substance use disorder rather than specific skills required to prescribe buprenorphine. This can likely be accomplished in <8 h and may have greater value early on in medical training, rather than later in a career when patterns and stereotypes are deeper ingrained (22). We believe this study provides preliminary evidence for the role of increasing exposure to a biopsychosocially focused SUD curriculum with a focus on decreasing the stigma associated with SUD.

The non-randomized design is a limitation to the study. Attendance was required for some resident and faculty learners, however others attended voluntarily and not all participants chose to complete the survey which may have led to a group that was predisposed to a positive attitude change. Another limitation is the limited geographic range of the trainings. It is possible that stigma and attitudes may differ depending on regional variations of historical experiences and rates of opioid abuse. Additionally, we used a validated survey for provider attitudes, however we added several questions related specifically to buprenorphine prescribing at the end of the survey, which may affect the validation. Another limitation is that we administered the post-test immediately after the training. In order to protect the privacy of our participants and due to follow-up data requirements from our funders, we collected the demographics separately from the survey data and so we were not able to do a sub-group analysis to see if there were differences in attitude changes based on the gender, level of training or specialty. Further research is needed to determine if the attitude shift is maintained over time and if there are subgroup changes in attitude changes based on level of training, specialty or gender.

## Conclusion

Increasing medical education around substance use disorders is a critical next step to decreasing stigma around this disease and improving access to treatment. It is likely that the full 8 or 24 h are not required to change attitudes. The previous, federally required training is not an evidenced based educational intervention. Now that the laws have changed to remove the requirement for a specific training larger, randomized controlled studies are needed to determine if our biopsychosocially focused, cased based approach, or if having a team of primary care clinicians direct the intervention, is the critical aspect for changing clinicians understanding and attitudes toward substance use disorders. Additionally, there should be consideration of including high yield elements from the buprenorphine waiver training as a routine requirement for undergraduate medical education to standardize the message received by clinicians in training.

## Data Availability Statement

The datasets presented in this study can be found in online repositories. The names of the repository/repositories and accession number(s) can be found at: https://app.box.com/s/vkqomvxqsey1d91dyp3tmbb0tp7704w8.

## Ethics Statement

The studies involving human participants were reviewed and approved by University of Rochester School of Medicine and Dentistry RSRB. Written informed consent for participation was not required for this study in accordance with the national legislation and the institutional requirements.

## Author Contributions

BS cleaned the data, analyzed, interpreted the baseline, and post-training data. EL, HR, and MS interpreted the data analysis and contributed to writing the manuscript. All authors read and approved the final manuscript.

## Conflict of Interest

The authors declare that the research was conducted in the absence of any commercial or financial relationships that could be construed as a potential conflict of interest. The handling editor MW declared a shared affiliation, though no other collaboration, with one or more authors HR, BS, and MS at the time of the review.

## Publisher's Note

All claims expressed in this article are solely those of the authors and do not necessarily represent those of their affiliated organizations, or those of the publisher, the editors and the reviewers. Any product that may be evaluated in this article, or claim that may be made by its manufacturer, is not guaranteed or endorsed by the publisher.
